# The multi-specific V_H_-based Humabody CB213 co-targets PD1 and LAG3 on T cells to promote anti-tumour activity

**DOI:** 10.1038/s41416-021-01684-4

**Published:** 2021-12-30

**Authors:** Carolyn J. Edwards, Angelica Sette, Carl Cox, Barbara Di Fiore, Chris Wyre, Daniela Sydoruk, David Yadin, Philip Hayes, Szymon Stelter, Phillip D. Bartlett, Miren Zuazo, Maria Jesus Garcia-Granda, Giovanni Benedetti, Stratoniki Fiaska, Neil R. Birkett, Yumin Teng, Carrie Enever, Hugo Arasanz, Ana Bocanegra, Luisa Chocarro, Gonzalo Fernandez, Ruth Vera, Bethan Archer, Isabelle Osuch, Martyna Lewandowska, Yasmin M. Surani, Grazyna Kochan, David Escors, James Legg, Andrew J. Pierce

**Affiliations:** 1 Crescendo Biologics Ltd., Meditrina Building, Babraham Research Campus, Cambridge, CB22 3AT UK; 2 Oncoimmunology Group, Navarrabiomed, Instituto de Investigaciones Sanitarias de Navarra (IdISNA) UPNA, Irunlarrea street, 3, 31008 Pamplona, Spain; 3 Department of Medical Oncology, Complejo Hospitalario de Navarra and Fundacion Miguel Servet, Instituto de Investigaciones Sanitarias de Navarra (IdISNA), Irunlarrea street, 3, 31008 Pamplona, Spain

**Keywords:** Antibody fragment therapy, Cancer immunotherapy, Drug development

## Abstract

**Background:**

Improving cancer immunotherapy long-term clinical benefit is a major priority. It has become apparent that multiple axes of immune suppression restrain the capacity of T cells to provide anti-tumour activity including signalling through PD1/PD-L1 and LAG3/MHC-II.

**Methods:**

CB213 has been developed as a fully human PD1/LAG3 co-targeting multi-specific Humabody composed of linked V_H_ domains that avidly bind and block PD1 and LAG3 on dual-positive T cells. We present the preclinical primary pharmacology of CB213: biochemistry, cell-based function vs. immune-suppressive targets, induction of T cell proliferation ex vivo using blood obtained from NSCLC patients, and syngeneic mouse model anti-tumour activity. CB213 pharmacokinetics was assessed in cynomolgus macaques.

**Results:**

CB213 shows picomolar avidity when simultaneously engaging PD1 and LAG3. Assessing LAG3/MHC-II or PD1/PD-L1 suppression individually, CB213 preferentially counters the LAG3 axis. CB213 showed superior activity vs. αPD1 antibody to induce ex vivo NSCLC patient T cell proliferation and to suppress tumour growth in a syngeneic mouse tumour model, for which both experimental systems possess PD1 and LAG3 suppressive components. Non-human primate PK of CB213 suggests weekly clinical administration.

**Conclusions:**

CB213 is poised to enter clinical development and, through intercepting both PD1 and LAG3 resistance mechanisms, may benefit patients with tumours escaping front-line immunological control.

## Background

Immune checkpoint inhibitors (ICIs) re-enable anti-tumour immunological activity and are demonstrating unprecedented efficacy in an increasing number of cancers since their introduction into clinical practice [1, 2]. The primary mechanism of action of most approved monoclonal antibody-based ICIs is to block the immunosuppressive interaction between programmed death ligand 1 (PD-L1) and programmed death-1 (PD1) [3]. PD-L1 is a type I transmembrane protein that belongs to the B7 family of ligands and is key to maintaining systemic immune tolerance and regulating inflammation [4]. PD-L1 expression on cancer cells plays multiple roles, including inhibiting interferon-induced apoptosis and accelerating proliferation [5, 6] as well as strong inhibition of anti-tumour T cell responses through binding to the receptor PD1 on T cells [7, 8]. Therefore, antibody blockade of PD-L1/PD1 interactions sensitises tumours to cytotoxic T cells while reactivating tumour-specific T cells by removing the T cell brakes [5, 9–11].

Nevertheless, a significant number of patients are intrinsically resistant to anti-PD-L1/PD1 mono blockade therapies. Indeed, these can account for up to 60–90% of treated patients depending on the type of cancer [12]. The reasons behind this intrinsic resistance are under active investigation. Recent findings suggest that acquired resistance to PD-L1/PD1 blockade is associated with systemic T cell dysfunctionality and enhanced co-expression of PD1 and lymphocyte activation gene 3 (LAG3) in T cells [13–15], consistent with the well-accepted role of PD1 and LAG3 in the exhaustion of tumour-infiltrating T cells [13, 15–18]. LAG3 is a type-I transmembrane protein phylogenetically related to CD4 molecules, which acts as an inhibitory immune checkpoint by binding major histocompatibility complex II (MHC-II) on the antigen-presenting cell. Additional ligands for LAG3 have been identified and include LSECtin [19], Galectin-3 [20], α-synuclein [21] and FGL-1 [22]. The synergy between PD1 and LAG3 has been observed for anti-PD1 and anti-LAG3 antibody combinations in preclinical tumour models [15, 23]. Most recently, the therapeutic benefit of combination αPD1/αLAG3 treatment in melanoma patients has been clinically validated [24, 25] including in a randomised phase 3 study [26]. T cells expressing LAG3 commonly also express PD1 [27], and these highly dysfunctional PD1+LAG3+ dual-positive T cells have been associated with resistance to conventional ICI therapies and hyperprogression [13, 14]. An antagonistic agent that would directly target these PD1/LAG3 double-positive cells may constitute an alternative for patients with high probabilities of failure to respond to conventional immunotherapies.

Humabody® V_H_ are single-domain antibodies originating from the Crescendo mouse. The mouse is a transgenic platform combining an engineered heavy chain locus with a broad range of human heavy chain V, D and J genes, a modified mouse Cγ1 gene and a complete 3’ regulatory region, in a background devoid of endogenous immunoglobulin expression [28]. The Crescendo mouse generates heavy chain-only antibodies with fully human V_H_ domains that can be derived from any of the range of human germline genes present in its translocus. These monovalent Humabody V_H_ can be amplified from the lymphoid tissue of immunised mice to generate libraries of target-specific domain antibodies. Each individual V_H_ moiety is approximately 12 kDa, considerably smaller than a conventional 150 kDa monoclonal IgG antibody, and V_H_ domains can be used on their own or can be joined to other V_H_ domains via peptide linkers to create multi-specific molecules. Such multi-specifics are designed to overcome some of the limitations of traditional IgG-based molecules in order to achieve therapeutic agents with improved characteristics such as greater tumour penetration due to smaller molecular size or differential target engagement [29].

Here we describe the generation and primary pharmacology of CB213, a novel asymmetric bispecific humabody to block signalling through both LAG3 and PD1. The selective binding of CB213 towards cells expressing both LAG3 and PD1 is designed to target the most dysfunctional tumour-specific T cells to reinvigorate T cell activity. CB213 is also formatted with V_HH_ targeting albumin for plasma half-life extension (HLE) and shows pharmacokinetic (PK) properties in cynomolgus macaques consistent with enabling clinical studies in human patients [30].

## Methods

### Generation of individual Humabody® V_H_

Mice with a background that is silenced for endogenous antibody expression (triple knock-out) were created as previously described [28]. These Crescendo mice have been shown to express a diverse repertoire of recombined antibody heavy chain-only sequences and show robust responses to immunisation. The selection and production of Humabody V_H_ have been previously described [28].

Crescendo mice were immunised with human LAG3 or human PD1 proteins. Lymphoid tissues from these mice were then used for RNA extraction and RT-PCR amplification of V_H_ domain antibody sequences. V_H_ sequence libraries were cloned into a vector for phage display panning selections. These selections enriched antigen-reactive Humabody® V_H_ and screening from the selected libraries delivered a diverse panel of sequences. To further investigate their properties V_H_ were cloned for expression in *Escherichia coli*. The proteins were purified via C-terminal histidine tags and screened for function. The first LAG3-binding V_H_ monomer identified is designated ‘LG204D9’. The finalised LAG3-binding V_H_ monomer is designated ‘LGB’ and differs from LG204D9 through site-directed mutagenesis of four amino acid residues to restore point mutants to a fully human homologous sequence. A separate finalised PD1 binding monomer V_H_ derived similarly is designated ‘PDB’.

### Assembly and expression of CB213 and multi-specific V_H_ constructs

Multiple tandem copies of a unit glycine/serine linker (‘GS’: Gly_4_Ser) between V_H_ components replace any intervening histidine tag sequences and provide spacing and molecular flexibility to format multiple V_H_ units in series. Two copies of the lead LAG3 monovalent V_H_, LGB were connected into a bivalent LGB-6GS-LGB sequence in this manner and then subsequently further formatted with the C-terminal addition of a PD1 binding, ligand-blocking monovalent V_H_ (PDB) with each V_H_ linked together by glycine/serine linkers to form the construct LAG3-LAG3-PD1. Plasma HLE was added by linking a human serum albumin (HSA) binding antibody fragment (V_HH_) to the C-terminus of LAG3-LAG3-PD1 via a glycine/serine linker resulting in the CB213 molecule: LAG3-LAG3-PD1-HSA. The predicted molecular weight of CB213 is 59.2 kDa.

CB213 was expressed in a microbial system and purified chromatographically. The integrity of purified CB213 was verified by size exclusion chromatography and mass spectrometric analysis. The samples were concentrated to 10 mg/ml and endotoxin depleted where required.

### Multi-specific target affinity/avidity measurements

Simultaneous bispecific binding to LAG3 protein (R&D Systems 2319-L3) and PD1 protein (R&D Systems 8986-PD) was determined using Dynamic Biosensors switchSENSE technology with dual-colour detection [31]. The antigens were covalently attached to the biosensor surface using the amine-coupling method, each antigen was linked to a different nanolever, with a red or green fluorescent tag. Binding of bispecific was measured to individual antigens alone (single binding) or where both antigens were present on the surface simultaneously (dual binding). Calculations are based on either 3- or 4-point dilution affinity measurements.

### LAG3/MHC-II functional disruption in vitro pharmacology reporter assay

Raji cells expressing MHC-II and Jurkat-LAG3 reporter cells were sourced from Promega (cat. CS194801). In our experience, the use of this commercially available well-defined reporter gene system gives more reproducible results than primary cell reactions from mixed lymphocyte cultures. Samples were diluted in assay buffer and a dilution series was prepared. Additional wells with assay buffer only were prepared. The diluted samples were then added to 384-well plates together with Jurkat-LAG3 cells (40,000 cells/well) and Raji cells (20,000 cells/well) and SED (staphylococcal enterotoxin D). The plates were incubated for 6 h at 37 °C in a 5% CO_2_ incubator. BioGlo detection reagent (Promega) was added, and the luminescence signal generated by the integrated NFAT response element reporter gene was measured on a BMG PHERAstar reader. The data is expressed as background subtracted (buffer-only control) signal in arbitrary relative light units (RLU). ‘Relatlimab’ is an analogue of relatlimab [32]. ‘aLAG3 17B4’ is a mouse monoclonal blocking antibody to human LAG3 (AdipoGen cat. AG20B001 2PF-C100). ‘DART’ is an analogue of tebotelimab/MGD-013 [33], a bispecific molecule that independently co-targets LAG3 and PD1.

### PD1/PD-L1 functional disruption in vitro pharmacology reporter assay

CHO cells stably expressing PD-L1 and anti-CD3 scFv (clone OKT3) were generated by Lipofection transfection and clonal selection. Jurkat cells expressing human PD1 were generated by electroporation of a plasmid encoding full-length PD1 into Jurkat pNL NFAT reporter cells (Promega). PD1 positive cells were sorted by FACS and expanded. CHO-PD-L1-OKT3 cells were plated at 10,000 cells/well in a 96 well plate overnight at 37 °C in a 5% CO_2_ incubator on the day prior to the reporter assay. Samples were diluted in assay buffer. Jurkat-PD1 NFAT luciferase reporter cells were added at a final density of 25,000 cells per well. The plates were incubated for 6 h at 37 °C in a 5% CO_2_ incubator. Nano-Glo detection reagent was added, and the luminescence signal was measured on a PHERAstar plate reader (BMG Labtech). The data are expressed as background subtracted (buffer-only control) signals in arbitrary RLU.

### Human patient ex vivo pharmacology studies

Blood samples were prospectively collected from a cohort of patients with locally advanced or metastatic NSCLC initiating treatment with ICI (nivolumab, pembrolizumab and/or atezolizumab). Clinical details and composition of the cohort under study are described in detail elsewhere [13, 15]. Briefly, eligible patients were ≥18 years of age and had all progressed on first-line platinum-based chemotherapy or concurrent chemoradiotherapy. A basal CT scan before the beginning of immunotherapy and a previous one was analysed. Exclusion criteria were previous immunotherapy treatment or the existence of synchronous neoplasms. Four millilitres of peripheral blood samples were obtained immediately prior to the infusion of the first cycle of immunotherapy.

Patient or healthy donor PBMCs were isolated by FICOL gradients immediately after the blood extraction. PBMCs were washed and T cells were isolated as described [15]. T cells were maintained in TexMACs medium (Miltenyi) until use. SC3-A549 cells were obtained as described [15] and maintained in RPMI medium complemented with 10% foetal calf serum (FCS), supplemented with penicillin and streptomycin. Co-cultures with T cells were carried out as described [15]. Briefly, T cells were added in complete RPMI medium without cytokines to reach a ratio of 2:1 A549-SC3:T cells.

The negative control treatment was a bispecific V_H_ that binds to the irrelevant targets hen egg lysozyme and mouse serum albumin (HEL-MSA). Blocking antibody/Humabody treatments were added at saturating concentrations of 100 nM, with the negative control at 171 nM. When the LAG3 bivalent V_H_ construct was tested it was used at 488 nM. Co-cultures were carried out for 2 days.

Cells were collected and washed twice in phosphate-buffered saline (PBS). Cells were blocked with PBS–20% FCS containing FcR blocking antibodies (1:200) for 15 min on ice followed by incubation in 50 µl of PBS-FCS-FcR buffer containing the appropriate antibody dilutions for 5 min on ice for surface staining. The antibodies used were: CD4-APC-Vio770 (clone M-T466, Miltenyi Biotec), CD3-APC (clone REA613, Milenyi Biotec), CD28-PECy7 (clone CD28.2, Biolegend), PD-1-PE (clone EH12.2H7, Biolegend), CD8-FITC (clone SDK1, Biolegend), LAG-3-PE (clone 11C3C65, Biolegend), LAG-3-PerCP-Cy5.5 (Clone 11C3C65, Biolegend).

After staining, cells were washed once with 1 ml of PBS and resuspended in 200 µl of PBS before either flow cytometry or intracellular staining. All steps were performed on ice. When required, surface staining was followed by intracellular staining using the BD TF buffer set. The following antibodies were used for staining: anti-Ki67-APC or pacific blue-conjugated antibodies (Clone ki67, BioLegend), AF648-CBL-B (G1 clone, Santa Cruz Biotech) and AF648-C-CBL (A-9 clone, Santa Cruz Biotech) antibodies. Flow cytometry was carried out with a BD FACS CANTO flow cytometer, recording all events for each sample. Data were analysed by the Flowjo software.

### CB213 cross-species target affinities

A FortéBio OCTET instrument was used to study the interaction between CB213 with human (R&D Systems 2319-L3) and recombinant cynomolgus monkey LAG3-Fc tagged proteins (R&D System, 8578-PD-050) and similarly for recombinant human and cynomolgus monkey Fc-tagged PD1 (R&D Systems: 1086-PD, 8578-PD-050), and for albumin from human (Sigma Aldrich A3782), cynomolgus monkey (Abcam ab184894) and mouse (Sigma Aldrich A3559). Murine his-tagged LAG3 (Acro Biosystems LA3-M52H5) and recombinant mouse-PD1-human-Fc chimaera (R&D Systems, 1021-PD-100) proteins were also assessed, as was IgG from human serum (Sigma Aldrich I2511).

Targets in sodium acetate pH 6.0 were coupled to AR2G biosensors by amine coupling using the amine reagent coupling kit (2nd generation, FortéBio). Binding was then analysed against CB213 at a range of predetermined concentrations (7-point 2-fold dilution series in 1× dPBS + 0.02% Tween + 0.1% BSA, with a top concentration of 250 nM) with an association phase of 200 seconds and a dissociation phase of 300 seconds. The kinetics and affinities of the binding interactions were modelled using a 1:1 binding model and calculated using the FortéBio Analysis Software version 8.1 or above.

### CB213 PK in cynomolgus macaques

To assess the PK of CB213, two female cynomolgus monkeys at Envigo CRS Ltd (Huntingdon, UK) were dosed with a single intravenous bolus injection of CB213 at 4 mg/kg via a saphenous vein, and blood samples were collected at pre-dose, 1, 2, 4, 8, 24, 48, 72, 120, 168, 216, 264, 312, 360, 408 and 504 h post-drug administration.

Serum samples were analysed on the Gyrolab Xplore immunoassay platform using biotinylated human LAG3 as a capture reagent and DyLight-650 labelled human PD1 as a detection reagent. CB213 concentrations in serum were measured at each timepoint. PK analysis of data was undertaken using PK Solver v2.0.

The in-life experimental procedures undertaken during the course of this study were subject to the provisions of the United Kingdom Animals (Scientific Procedures) Act 1986 Amendment Regulations 2012 (the Act). The number of animals used was the minimum consistent with scientific integrity and regulatory acceptability, consideration having been given to the welfare of individual animals in terms of the number and extent of procedures to be carried out on each animal.

### CB213 anti-drug antibodies (ADAs) in cynomolgus macaques

An ADA assay was developed and performed using the Gyrolab immunoassay platform. The format is a direct immunoassay in which CB213 is immobilised by LAG3 biotinylated antigen. The analyte (ADA) bound to CB213 is then detected by anti-human Ig(L) labelled with Alexa Fluor 647.

### CB213 in vivo pharmacology in a syngeneic colorectal cancer (CRC) tumour model

MC38 colon carcinoma cells were supplied by CrownBio and were maintained in vitro with DMEM medium supplemented with 10% foetal bovine serum at 37 °C in an atmosphere of 5% CO_2_ in air. Transgenic hPD1/hLAG3 HuGEMM mice (C57BL/6 background) were supplied by Crown Bioscience or designated subsidiaries and were engineered using CRISPR/Cas9 technology to express the human PD1 and human LAG3 extracellular domain in the mouse PD1 and LAG3 loci, respectively.

hPD1/hLAG3 HuGEMM mice (*N* = 10 mice per group, consistent with sample sizes typical for in vivo assessments of immunomodulators) were inoculated subcutaneously in the right rear flank region with MC38 tumour cells (1 × 10^6^) in 0.1 ml of PBS for tumour development. The day of cell inoculation was denoted as day 0. Mice were randomised at a mean tumour size of approximately 80–120 mm^3^ based on a ‘Matched distribution’ method/‘Stratified’ method (StudyDirectorTM software, version 3.1.399.19)/randomised block design. Fifty mice were enrolled in the efficacy study allocated across 5 study groups. Dosing was every other day (Q2D) with CB213, nivolumab (CrownBio) or HEL-MSA as a negative control. HEL-MSA is a bivalent Humabody consisting of a hen egg lysozyme-binding V_H_ [34] connected to a V_H_ designed to bind mouse serum albumin for plasma HLE [28] by a 6GS linker.

Mouse protocols and any amendment(s) or procedures involving the care and use of animals were reviewed and approved by the Institutional Animal Care and Use Committee of CrownBio prior to execution. During the study, the care and use of animals were conducted in accordance with the regulations of the Association for Assessment and Accreditation of Laboratory Animal Care. The study was not conducted in a blinded fashion.

### Flow cytometry from syngeneic mouse tumours

At study termination, tumour samples from the Humabody negative control group and the CB213 10 mg/kg group were collected, minced into fine pieces and transferred into C-tubes (Miltenyi) containing 2.5 ml digestion buffer (Tumour dissociation kit) and disaggregated in a Miltenyi GentleMACS machine. Cells were filtered through 70 μm mesh, centrifuged and washed twice with RPMI 1640. Cells were counted and Fc blocked (purified rat anti-mouse CD16/32 BD Fc block: BD 553141) before staining. Tumour cells were stained with fluorochrome-conjugated antibodies to mCD45, mCD4, mCD8, hPD1 (Biolegend: Cat. 103132, 100406, 100708, 329918, respectively) and to mCD3, hLAG3 (BD: Cat. 740268, 565716).

## Results

### Multi-specific V_H_ constructs promote high avidity molecular interactions when co-targeting PD1 and LAG3

**Fig. 1:**
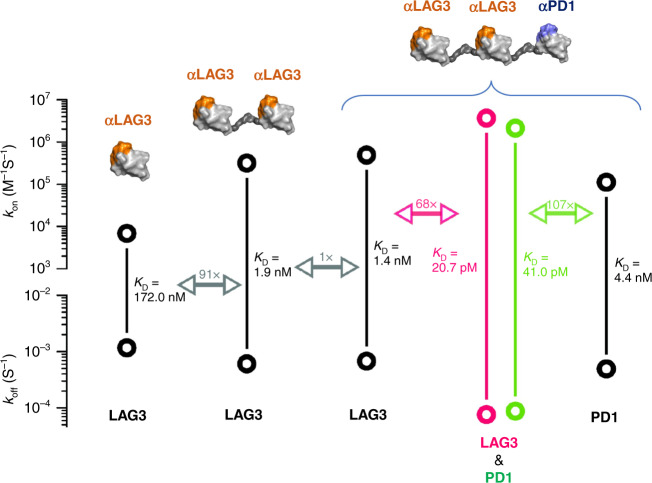
Binding constants and *K*_D_ values. Binding constants were assessed for various V_H_ constructs (illustrated on top) against specific molecular targets (listed at the bottom). Bold circles show measured *K*_on_ and *K*_off_ values with calculated *K*_D_ values indicated. Fold change in *K*_D_ values shown by double-headed arrows. Values are the means of three measurements.

Our initial monovalent V_H_ Humabody targeting LAG3, LG204D9, was generated and showed a promising level of affinity to the target of 172 nM. We sought to improve this binding by reformatting the Humabody as a bivalent construct with two copies of the LAG3-binding V_H_ separated by a flexible 6-copy glycine/serine linker domain. The bivalent format improved the binding to LAG3 by approximately two orders of magnitude to 1.9 nM (Fig. 1). Next, we added a third V_H_ adding in PD1 targeting to the C-terminus of the LAG3 bivalent V_H_ molecule again using a flexible glycine/serine linker region and taking advantage of the opportunity afforded by the additional molecular biology manipulations involved to revert four non-human germline amino acids in LG204D9 to fully human sequences. The resulting LAG3-LAG3-PD1 construct maintained or even slightly improved affinity to the LAG3 target with a *K*_D_ of 1.4 nM. Next, we assessed the binding affinity of LAG3-LAG3-PD1 to the PD1 target, which was 4.4 nM and we considered this to be acceptable for further drug development purposes. We further assessed the capacity of the multi-specific to simultaneous engage LAG3 and PD1 co-presented on a solid surface finding a nearly two orders of magnitude further improvement and validating our approach of using multi-specific V_H_ constructs to drive high avidity interactions with multiple targets (20.7 pM to LAG3 and 41.0 pM to PD1, Fig. 1).

### The multi-specific LAG3-LAG3-PD1 V_H_ construct promotes ex vivo NSCLC patient T cell proliferation

**Fig. 2:**
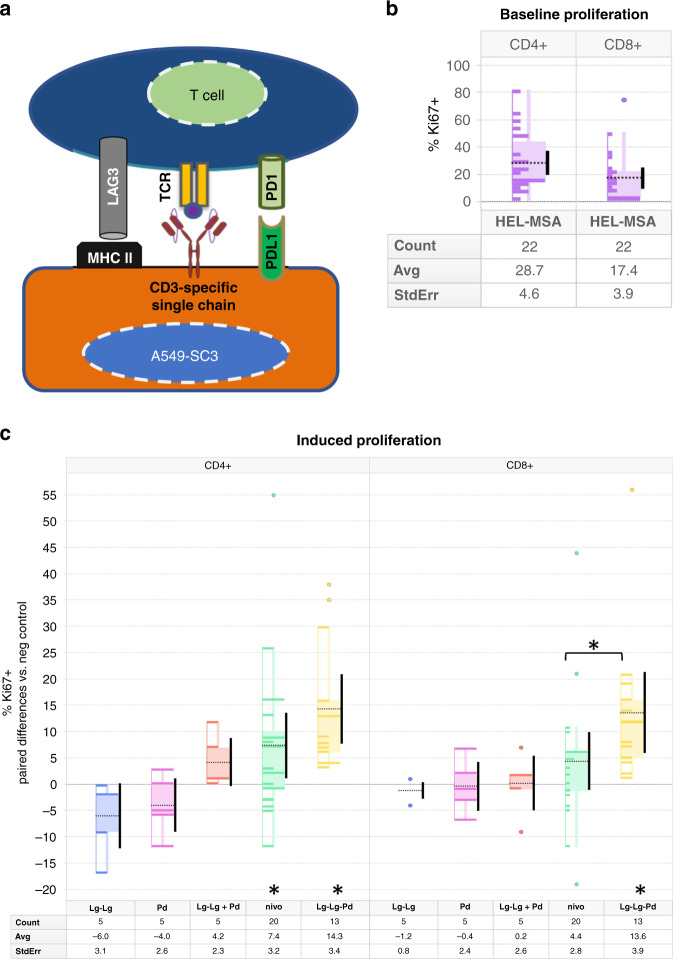
Human ex vivo T cell proliferation. **a** Overview of the tumour cell/T cell assay system. A549-SC3 cells [15] engage the TCR on T cells via a single chain αCD3 antibody. MHC-II and PD-L1 expressed on A549-SC3 cells suppress T cell proliferation by engaging LAG3 and PD1, respectively. **b** Baseline T cell proliferation with negative control treatment. Percent proliferation as shown by Ki67 positivity relative to total CD4+ and CD8+ T cell populations with HEL-MSA on board in ex vivo blood samples from 22 patients. Boxes represent quartiles, the dotted black line is the mean value, the solid vertical bar is the 95% confidence interval of the mean. ‘Count’ is the number of patients. **c** Proliferation of NSCLC patient T cells after ex vivo stimulation with A549-SC3 cells in the presence of various potential checkpoint modulators: Lg-Lg: two Lag3-binding V_H_ connected by a glycine/serine linker; Pd: one PD1-binding V_H_; Lg-Lg + Pd: Lg-Lg and Pd co-administered; nivo: nivolumab analogue mAb; Lg-Lg-Pd: LAG3-LAG3-PD1 triple V_H_ construct. All values represent the patient-specific paired difference in %Ki67+ T cells with respect to the negative control V_H_ construct HEL-MSA for each given patient. Boxes represent quartiles, the dotted black line is the mean value, the solid vertical bar is the 95% confidence interval of the mean. ‘Count’ is the number of patients. Asterisk indicates *P* > 0.05 by two-tailed Wilcoxon signed-rank test vs. negative control; asterisk above square bracket shows *P* > 0.05 by two-tailed unpaired Mann–Whitney test for nivolumab vs. LAG3-LAG3-PD1 in CD8+ T cells.

We wanted to test the capacity of V_H_ constructs directed against PD1, LAG3 or a multi-specific V_H_ construct targeting both PD1 and LAG3 simultaneously to promote proliferation of T cells in a clinically relevant context where both PD1- and LAG3-mediated immunosuppressive mechanisms were present. Accordingly, we obtained peripheral T cells from NSCLC patients initiating ICI following front-line chemotherapy and assessed proliferation ex vivo using a T cell stimulator cell line, described elsewhere [15], that models recognition of cancer cells by T cells in the presence of inhibitory signalling through both PD1 and LAG3 axes. Briefly, this human A549 lung adenocarcinoma derived line expresses a membrane-bound anti-CD3 single-chain antibody (A549-SC3 cells) for T cell engagement and both MHC-II and PD-L1 which can provide inhibitory interactions through LAG3 and PD1 respectively on the T cells (Fig. 2a). Enhancement of T cell proliferation was measured by quantifying Ki67 expression relative to ex vivo treatment of these patient-derived T cells with the negative control HEL-MSA Humabody. We observed considerable inter-patient variability in levels of baseline cellular proliferation in this system (Fig. 2b), nevertheless, the relative effects of different ex vivo treatments showed consistent differences. As shown in Fig. 2c, neither a LAG3-LAG3 bivalent V_H_ construct, nor a single V_H_ targeting PD1 were able to significantly enhance either CD4+ or CD8+ T cell proliferation, nor was a combination treatment of the bivalent LAG3-LAG3 V_H_ and the PD1 V_H_ admixed together. A full-length triple V_H_ LAG3-LAG3-PD1 construct, however, was able to promote significant T cell proliferation in both CD4+ and CD8+ lineages, supporting the multi-specific avidity therapeutic hypothesis. A commercially available analogue of nivolumab also supported T cell proliferation in this experimental context, particularly among CD4+ T cells, however, to a lesser extent than the LAG3-LAG3-PD1 V_H_ construct.

### CB213 structure and cross-species binding affinities

**Fig. 3:**
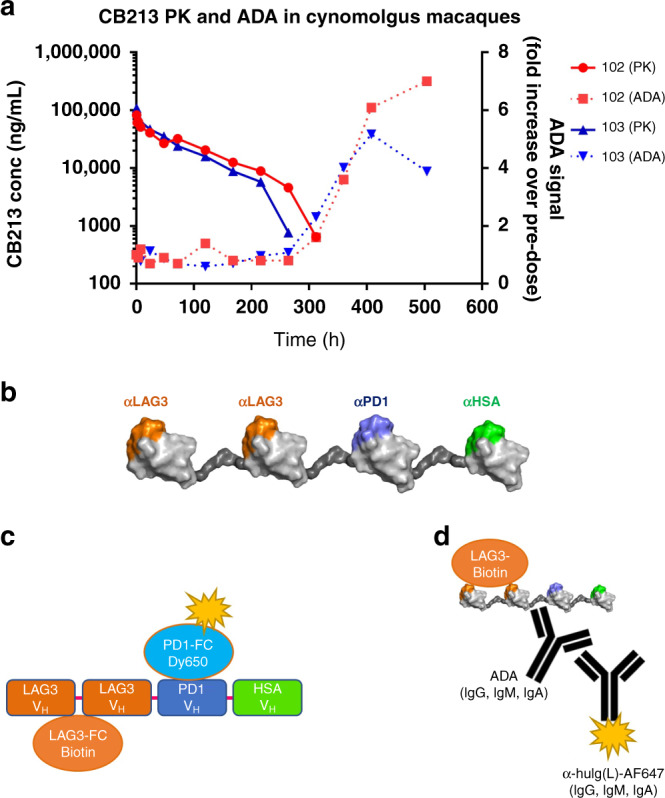
CB213 NHP pharmacokinetics and anti-drug antibodies. **a** CB213 PK and ADA in two female cynomolgus macaques: animals 102 and 103. **b** Cartoon of CB213 structure. A Schematic of CB213 is shown. Individual V_H_ are shown as globular domains, with stylised glycine/serine linkers intervening in dark grey. The diagram is not to scale. **c** Schematic of the MSD-based CB213 PK bridging assay. Molecules that can simultaneously bind to LAG3 and to PD1 are detected. **d** Schematic of the MSD-based ADA assay. Molecules that can bind to CB213 when bound to LAG3 that contain an IgG light chain are detected.

CB213 is based upon the LAG3-LAG3-PD1 triple V_H_ construct with the addition of a C-terminal albumin binding V_HH_ fragment to confer prolonged PK exposure and a greater degree of tissue penetration [29]. A schematic of the finalised structure of CB213 is shown in Fig. 3b. The binding of CB213 to recombinant and/or purified targets LAG3, PD1 and albumin was assessed across three species: human, cynomolgus macaque and mouse. CB213 bound with high affinity to LAG3 and PD1 from both human and cynomolgus macaque but exhibited no detectable binding to the mouse homologues of either of these proteins. Significant affinity to serum albumin from both human and cynomolgus macaque was also observed, with weaker but still significant affinity to mouse albumin as well. The *K*_D_ measurements for CB213 across these targets and species, assessed as triplicate measurements, are summarised in Table 1.

**Table 1:** Affinity of CB213 to cross-species targets (*K*_D_).

Species	LAG3	PD1	Albumin
Human	2.82 ± 0.33 nM	3.87 ± 0.28 nM	7.28 ± 0.06 nM
Cynomolgus macaque	4.32 ± 0.52 nM	5.53 ± 0.34 nM	6.70 ± 0.08 nM
Mouse	Not detected	Not detected	148 ± 1.5 nM

### CB213 in vitro functional suppression of LAG3/MHC-II interactions

**Fig. 4:**
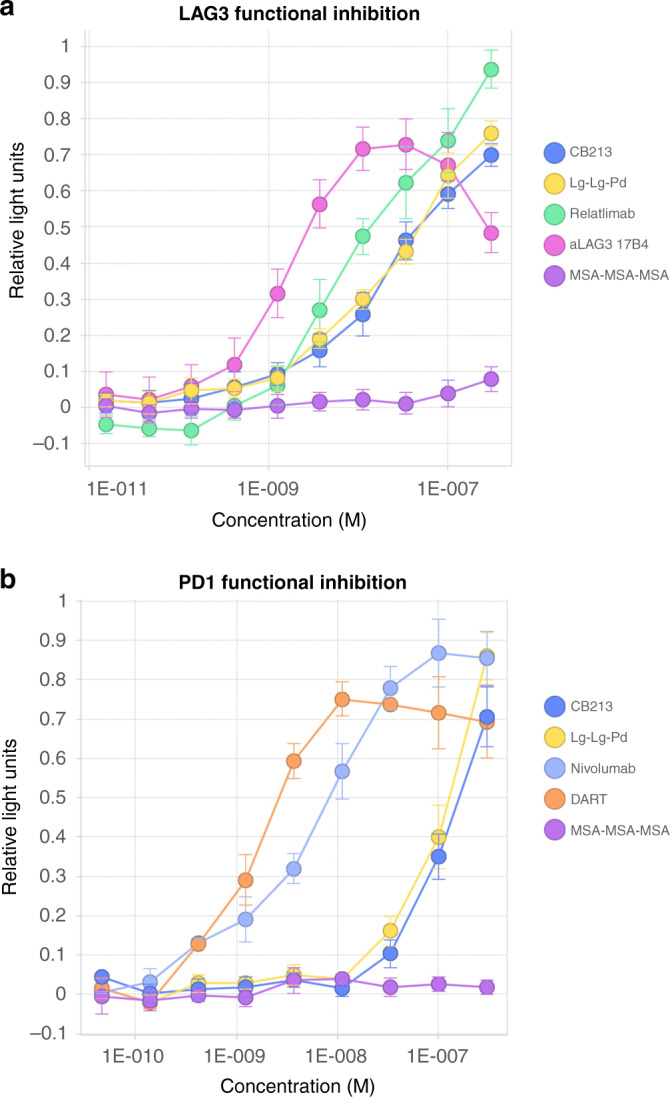
Functional inhibition of LAG3/MHC-II and PD1/PD-L1 interactions. Cell-based assessment of T cell activation measured by induction of an NFAT response element-driven luminescent reporter gene. Relative light units (arbitrary scale) are reported. **a** Relief of LAG3/MHC-II-mediated suppression. Standard errors of the mean are indicated. Approximate half-maximal stimulation concentrations: CB213: 20 nM, Lg-Lg-Pd (LAG3-LAG3-PD1): 22 nM, relatlimab: 9.7 nM, aLAG3 17B4 (anti LAG3 mouse monoclonal): 1.6 nM. MSA-MSA-MSA is three interlinked V_H_ recognising mouse serum albumin as a negative control. **b** Relief of PD1/PD-L1-mediated suppression. Standard errors of the means are indicated. Approximate half-maximal stimulation concentrations: CB213: 105 nM, Lg-Lg-Pd (LAG3-LAG3-PD1): 115 nM, nivolumab: 6 nM, DART (MGD-013): 1.4 nM. MSA-MSA-MSA is three interlinked V_H_ recognising mouse serum albumin as a negative control.

We utilised a co-culture system based on the interaction between MHC-II on Raji cells and LAG3 molecules constitutively presented on the surface membrane of Jurkat cells. These cells are engineered to activate an agonistic signal through the engagement of TCR with a superantigen (SED), which results in the activation of a downstream NFAT-RE-Luc2 reporter gene. This activation is inhibited by binding of MHC-II to LAG3 present on Jurkat cells where disruption of the LAG3/MHC-II interaction relieves the inhibitory effect resulting in a luminescent signal. We found that both the LAG3-LAG3-PD1 multi-specific V_H_ construct and CB213 were able to relieve the suppressive signal to a similar extent, with half-maximal stimulation at 22 and 20 nM, respectively, similar to that found with an analogue of relatlimab at 9.7 nM (Fig. 4a).

### CB213 in vitro functional suppression of PD1/PD-L1 interactions

A similar co-culture system was used to interrogate PD1/PD-L1 functional disruption, except in this case T cell stimulation was provided by CHO cells stably expressing both an αCD3 antibody and human PD-L1. Jurkat cells engineered to constitutively express PD1 quantify stimulation suppressed by the PD-L1/PD1 interaction, again using an integrated NFAT-RE-Luc2 reporter gene. LAG3 has not been provided to these Jurkat reporter cells, and consistent with avidity measurements the potency of CB213 for reversal of PD1/PD-L1-mediated suppression in the absence of LAG3 was greatly reduced, with half-maximal stimulation at 105 nM, again similar to that found with LAG3-LAG3-PD1 at 115 nM. In contrast, for example, an analogue of nivolumab provided half-maximal relief of PD1/PD-L1 suppression in this system at a concentration of 6 nM and a DART®-based molecule showed half-maximal suppression at 1.4 nM (Fig. 4b).

### CB213 PK in cynomolgus macaques

Having established that CB213 engages all three component targets, LAG3, PD1 and serum albumin across protein homologues from *Macaca fascicularis*, we sought to determine the PK properties of the molecule in this non-human primate (NHP).

Non-compartmental PK properties determined following single bolus dose injection in each of two female animals (Animal 102 and Animal 103, respectively) are summarised in Table 2 and shown in Fig. 3a. We found that CB213 has an estimated half-life in NHP of 66.8 ± 4.8 h (*n* = 2) when dosed at 4 mg/kg intravenously in cynomolgus macaque, which suggests weekly dosing of human patients in clinical studies may be feasible to achieve suitably sustained target coverage across the dosing interval.

**Table 2:** CB213 main pharmacokinetic parameters in cynomolgus macaques.

Parameter	Unit	Animal 102	Animal 103	Mean	SD	%CV
*T*_1/2_	Hours	70.3	63.4	66.8	4.83	7.2
*C*_max_	ng/ml	82,964	109,203	96,083	18,554	19.3
AUC∞	ng/ml*h	6,151,174	5,682,838	5,917,006	331,164	5.6
CL	(mg/kg)/(ng/ml)/h	6.50E−07	7.04E−07	6.77E−07	3.79E−08	5.6
Vss	(mg/kg)/(ng/ml)	6.88E−05	6.03E−05	6.46E−05	5.98E−06	9.3

*T*_*1/2*_ half-life, *C*_*max*_ maximum plasma concentration, *AUC∞* area under plasma concentration curve from time zero to infinity, *CL* clearance, *Vss* volume of distribution at steady state.

The PK profile for the two test animals demonstrated non-linear clearance beyond 264 h and 216 h in each case. As expected for a fully human biologic infused into non-human primates [35, 36] an ADA response was observed in both animals beyond 312 h, and accordingly, tabulated PK parameters have been calculated excluding data from time points post 264 h for the first animal and post 216 h for the second. The appearance of ADA has a good temporal correlation with accelerated PK clearance of CB213 at these later time points (Fig. 3a) consistent with ADA-mediated accelerated drug clearance.

### CB213 in vivo syngeneic mouse pharmacology

**Fig. 5:**
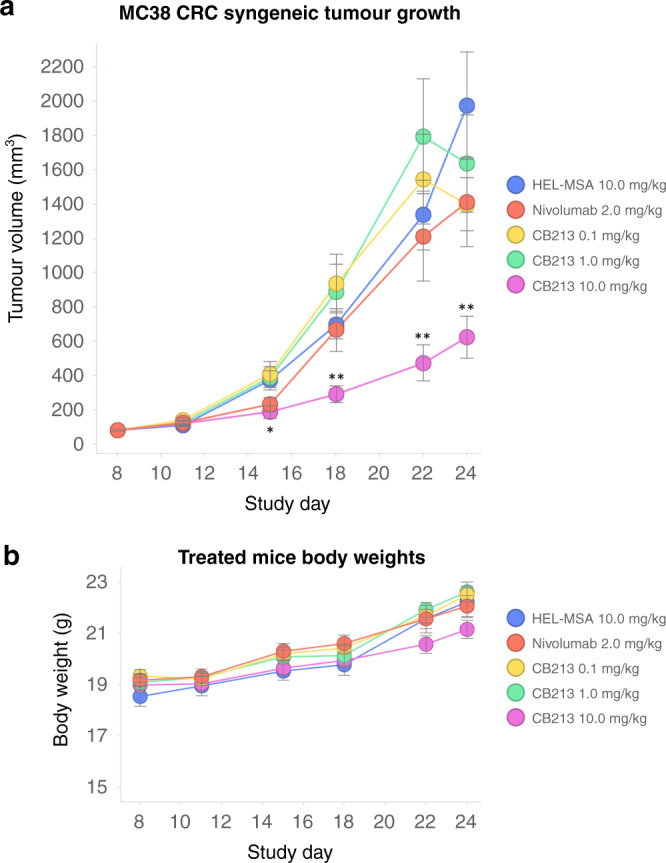
CB213 in vivo syngeneic mouse pharmacology. **a** Effect of CB213 on tumour growth: mice (*N* = 10 per group) were treated with CB213 10, 1 and 0.1 mg/kg, or control Humabody HEL-MSA (negative control) 10 mg/kg in all cases Q2D for 9 doses; or Nivolumab 2 mg/kg BIW for 3 doses. Means for each group are plotted. Error bars represent SEM. Statistical significance by two-tailed Mann–Whitney *U*-test: *P < 0.05, **P < 0.01 when compared to negative control. **b** Animal body weights over time. Means for each group are plotted. Error bars represent SEM.

Mouse MC38 CRC tumour cells were implanted into hPD1/hLAG3 transgenic mice and treated with either of three different concentrations of CB213, the anti-PD1 antibody nivolumab [37], or a negative control Humabody (HEL-MSA). CB213 at 10 mg/kg Q2D resulted in 64% tumour growth inhibition (TGI), whereas in this experimental system neither lower doses of CB213 nor nivolumab showed appreciable levels of TGI (Fig. 5a). The various mouse treatments were all well-tolerated with no decreased body weight over the duration of the study (Fig. 5b). The relatively high dosing levels of CB213 used in this syngeneic mouse in vivo pharmacology study vs. levels that may be anticipated for human dosing are to compensate to some extent for reduced albumin-mediated systemic drug exposure in the mouse since the affinity of CB213 to mouse serum albumin is considerably less than the affinity of CB213 to human serum albumin (Table 1).

**Fig. 6:**
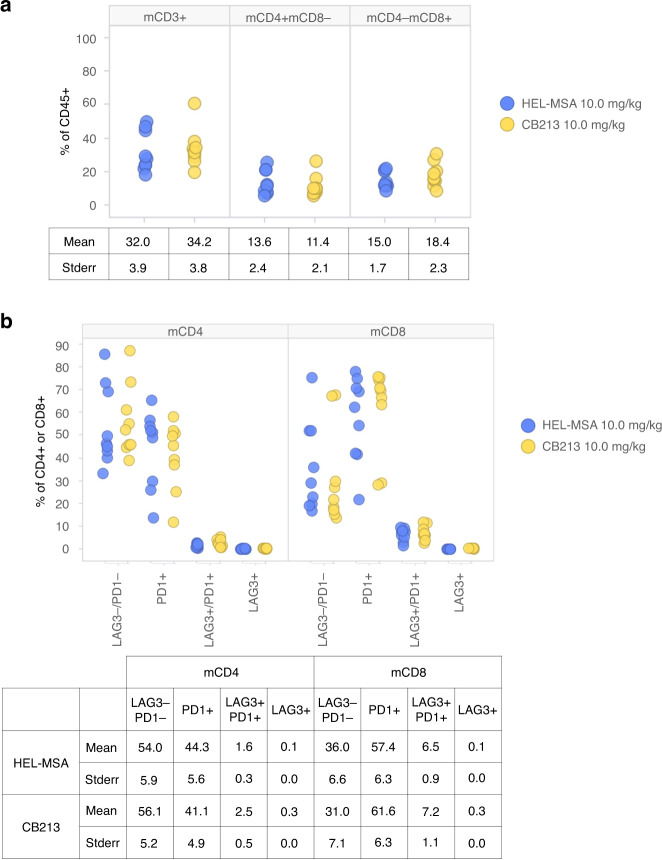
CB213 in vivo syngeneic mouse immunophenotyping. End-of study in vivo pharmacology syngeneic tumours disaggregated and analysed by flow cytometry. Treatments were HEL-MSA (negative control) or CB213 dosed 10 mg/kg. Datapoints represent single tumours from individual animals. **a** Tumour-infiltrating lymphocytes as a percentage of leucocytes (CD45+) in disaggregated tumours. **b** Expression of PD1 and/or LAG3 on CD4+ or CD8+ tumour-infiltrating lymphocytes, respectively.

Flow cytometric analysis of disaggregated end-of-study MC38 tumours showed significant levels of tumour-infiltrating lymphocytes (TILs) that were not drug dependent (Fig. 6a). Most TILs from CD4+ lineages either expressed neither PD1 nor LAG3 or expressed PD1 alone with only a small number PD1+/LAG3+ cells (Fig. 6b). CD8+ lineages, however, showed both an increase in PD1+ cells and PD1+/LAG3+ double-positive cells relative to CD4+ lineages (*P* > 0.01 in both cases by Wilcoxon signed-rank test). Very few TILs from either CD4+ or CD8+ lineages expressed LAG3 alone without also expressing PD1.

## Discussion

Recently disclosed results of Phase 3 RELATIVITY-047 study have provided clinical validation for intercepting immunosuppressive signalling on T cells through PD1 and LAG3 by co-dosing two monoclonal antibody therapeutics, nivolumab and relatlimab, respectively [26]. The additional efficacy from the combination approach carries with it increased toxicity as well, with a near doubling in grade 3/4 adverse events (9.7% for nivolumab vs. 18.9% for nivolumab plus relatlimab). Our goal was to create a novel multi-specific therapeutic that would relieve the immunological suppression primarily on T cells expressing both PD1 and LAG3, rather than on all T cells expressing PD1. It is hoped that this approach will confer a clinical safety advantage while maintaining or indeed improving efficacy.

CB213 is a novel Humabody with four interlinked V_H_ domains, two of which engage LAG3, and one of which engages PD1. This LAG3 orientated stoichiometry supports an avidity of binding ensuring PD1 is bound primarily when LAG3 is also bound. While in vitro pharmacology studies involving single-target expression of LAG3 or PD1 (Fig. 4) show more potent alleviation of immunological suppression from lower concentrations of relatlimab or nivolumab vs. CB213 this may nevertheless not translate into better clinical efficacy from the single-target agents due to T cells acquiring resistance to monotherapies by co-expression of PD1 and LAG3.

We have illustrated how this focussed T cell-directed intervention can potentially show additional pharmacology beyond monotherapy ICI both in a syngeneic mouse tumour in vivo pharmacology model and in ex vivo studies interrogating T cells from treatment-experienced NSCLC patients, supporting clinical development of CB213 in patients with tumours progression following first-line treatment. The fourth V_H_ domain of CB213 that binds HSA supports enhanced biodistribution and extended plasma half-life where based on the observed PK in cynomolgus macaques we anticipate either a weekly or potentially biweekly schedule of clinical administration depending upon tolerability.

Humabody-based therapeutics have shown preclinical evidence of superior tumour penetration vs. traditional monoclonal antibodies [29], likely due to smaller physical size and potentially also tissue partitioning aided by both active and passive transport mediated by albumin binding [30]; these findings await evaluation in human patients. A Humabody-based therapeutic that has agonist activity against CD137 (4-1BB, HGNC:TNFRSF9) continent upon expression of prostate-specific membrane antigen in the tumour microenvironment, CB307, has recently begun clinical studies [38] and may provide the first clinical evidence of this hypothesis. In this regard, it will be interesting to compare and contrast Humabodies such as CB307 that are multi-specific for both tumour and T cells targets with those such as CB213 that double target T cell antigens.

## Supplementary information

Reproducibility checklist
